# Endemic Circulation of European Bat Lyssavirus Type 1 in Serotine Bats, Spain

**DOI:** 10.3201/1408.080068

**Published:** 2008-08

**Authors:** Sonia Vázquez-Morón, Javier Juste, Carlos Ibáñez, Eduardo Ruiz-Villamor, Ana Avellón, Manuel Vera, Juan E. Echevarría

**Affiliations:** * Instituto de Salud Carlos III, Majadahonda, Madrid, Spain; † Centro de Investigación Biomédica en Red de Epidemiología y Salud Pública (CIBERESP), Spain; ‡ Estación Biológica de Doñana CSIC, Sevilla. Spain; § Laboratorio Central de Veterinaria de Santa Fe, Granada, Spain

**Keywords:** lyssavirus, bats, surveillance, rabies, dispatch

## Abstract

To determine the presence of European bat lyssavirus type 1 in southern Spain, we studied 19 colonies of serotine bats (*Eptesicus isabellinus),* its main reservoir, during 1998–2003. Viral genome and antibodies were detected in healthy bats, which suggests subclinical infection. The different temporal patterns of circulation found in each colony indicate independent endemic circulation.

The serotine bat (*Eptesicus serotinus*) is considered the main reservoir for European bat lyssavirus type 1 (EBLV1). Recently, southern Iberian populations of this species have been classified as *E. isabellinus* (*1*), a bat species previously known to exist only in North Africa.

In 1989, 5 EBLV1-infected serotine bats were found dead during a survey of natural colonies in Huelva (Andalusia). The prevalence of EBLV1 antibodies was up to 20% among completely healthy bats that were recaptured 1 year later, providing the first direct evidence of the survival of serotine bats after EBLV1 infection (*2*). Furthermore, high seroprevalence and presence of viral RNA in the oral cavity and bloodstream of different lyssavirus species have been reported in healthy bats captured in natural colonies in which the numbers of deaths have not increased (*3*–*5*), showing direct evidence of subclinical or asymptomatic disease after viral infection. Even viral RNA and antigens have been detected in the brains of healthy captive bats (*6*). However, reports of experimental lyssavirus transmission have drawn discrepant conclusions (*7*–*9*).

## The Study

During 1998–2003, a total of 1,030 *E. isabellinus* from 19 colonies located in the provinces of Huelva, Seville, and Granada (Andalusia), in southern Spain, were sampled. We focused on this region because all EBLV1 cases reported in Spain were from southern Spain. Distances between colonies varied from 0.5 to 317 km.

Bats were captured mainly in so-called maternity colonies made up of adult females and young of both sexes. Each animal was banded and assessed for sex, age, size, and weight. A total of 150 (14.5%) were recaptured. The frequency of repeated captures reached 22.7% in individual bats from 3 colonies that were sampled in all 6 years of the study. All recaptured bats were always found in the same colony in which they were first marked.

Viral RNA was detected in 34 (2.8%) of 1,226 oropharyngeal swab specimens from 33 bats of 8 colonies (1 bat was resampled after a 1-week interval). One bat that tested positive was recaptured and tested negative in a followup sampling effort. Positive results were identified as EBLV1 by direct sequencing as previously described (*4*,*10*). Samples for reverse transcription–PCR were stored in a buffer that was designed for RNA preservation but was not suitable for keeping the virus viable for isolation on cell culture.

A total of 626 plasma samples were tested for EBLV1-specific antibodies by using a modification of the rapid fluorescent focus inhibition test (*11*), but 77 (12.3%) of them were found to be toxic to cells. EBLV1 antibodies were found in 51 (9.3%) of the remaining 549 from 13 colonies. Only 2 of 22 samples showing viral RNA in the oral cavity were antibody positive (Table). Thirteen antibody-positive bats were recaptured in a healthy condition in the following campaigns.

**Table:** European bat lyssavirus type 1 in *Eptesicus isabellinus* bats, Spain, 1999–2003*

Type of testing	RT-PCR	RFFIT
Total captures	1,226	626
No. colonies	19	13
Total no. (%) positive	34 (2.8)	51 (9.3)
1998		
No. captures	164	151
No. (%) positive	4 (2.4)	10 (6.6)
1999		
No. (%) captures	161	90
No. positive	4 (2.5)	5 (5.6)
2000		
No. captures	204	128
No. (%) positive	16 (7.8)	12 (9.4)
2001		
No. captures	209	96
No. positive	0	4 (4.2%)
2002		
No. captures	287	100
No. (%) positive	10 (3.4)	5 (5.0)
2003		
No. captures	201	54
No. positive	0	0

*Results obtained during active surveillance. RT-PCR, reverse transcription–PCR, RFFIT, rapid fluorescent focus inhibition test.

The temporal pattern of circulation was completely different in each colony (Appendix Table). Only 1 colony showed continuous circulation from 1998 through 2002.

**Figure 1:**
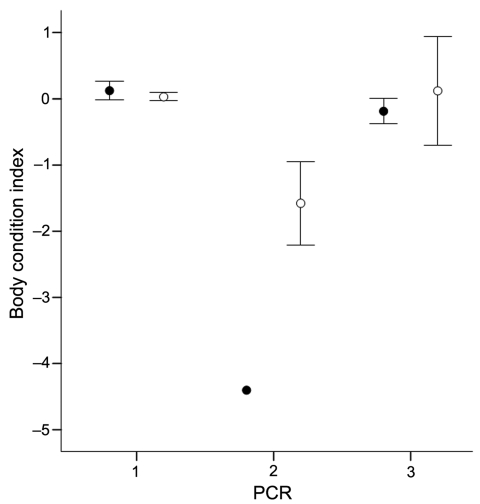
Relationship between body condition index (mean ± standard error) and diagnosis of European bat lyssavirus type 1 by reverse transcription PCR. Males and females are represented as filled and open circles, respectively. 1, only negative in oropharyngeal swab and brain specimens (● n = 49; ○ n = 225); 2, positive in oropharyngeal swab and brain specimens (● n = 1; ○ n = 4); 3, positive in oropharyngeal swab but negative in brain specimen (● n = 3; ○ n = 4).

Brains were obtained from 20 bats of the same colony that had been studied previously (*4*). Differences in body condition between noninfected bats, bats with positive oropharyngeal swabs only, and bats with positive oropharyngeal and brain specimens were tested by an analysis of variance; index of body condition was the dependent variable. Individual body condition was expressed as the residuals from an analysis of covariance with an optimized design, including body mass as dependent variable; forearm length as a covariate; and sex, age, and year as fixed factors. We found a significant negative association (Figure 1) between RNA presence and body condition (*F* = 11.78; degrees of freedom = 2, 281; p<0.001, n = 292). Post hoc tests indicated that only bats whose brains tested positive had a significantly worse body condition (Tukey honestly significant differences [HSD] test –2.33, p<0.001, and HSD –2.27, p<0.001, respectively). Differences in body condition between oropharyngeal swab–negative and oropharyngeal swab–positive bats were not significant (Tukey HSD 0.57, p = 0.99).

**Figure 2:**
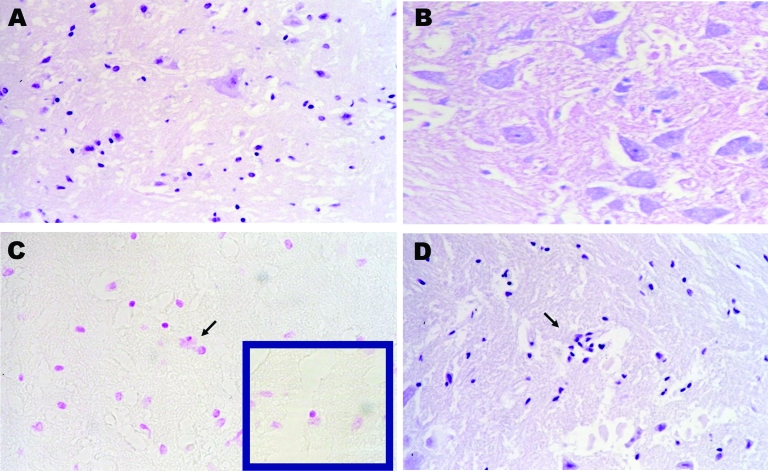
Pathologic images obtained from a carcass of Eptesicus isabellinus. The bat was captured while flying but died during handling. Brain specimen was positive for lyssavirus antigens by immunofluorescence and for European bat lyssavirus 1 RNA by reverse transcription–PCR. A) Neural degeneration in brain by hematoxylin and eosin stain (H&E); magnification ×400. B) Negative Seller stain in spinal cord indicating the absence of Negri bodies; magnification ×400. C) Positive Feulgen reaction in brain, glial cell neurophagia; magnification ×200. D) Focal proliferation of glial cells by H&E; magnification ×100.

Brain, cerebellum, and spinal cord from 1 bat carcass with natural EBLV1 infection were studied by histopathologic and histochemical techniques. The bat was captured while flying but died during manipulation, which is an extremely rare event. The brain and oropharyngeal swab were positive for EBLV1 by reverse transcription–PCR, and the brain smear was also positive by immunofluorescence. Histopathologic studies showed moderate neuronal degeneration characterized by neuronal hyperchromatosis, chromatolysis, and satellitosis (Figure 2, panel **A**). The Sellers stain results were negative, proving the absence of Negri bodies (Figure 2, panel **B**). The occasional presence of basophilic intracytoplasmic structures in neurons located in the cerebral cortex that underwent necrobiosis was interpreted as a neurophagic process that was taking place in the glial cells because these inclusions were shown to be positive by the Feulgen reaction (Figure 2, panel **C**), whereas the Negri bodies have a strongly acidophilic nature. Moderate gliosis without perivascular infiltration was observed (Figure 2, panel **D**).

## Conclusions

Despite the fact that serotine bats (*E. serotinus* and *E. isabellinus*) are the most frequently involved species in cases of bat lyssavirus exposure in humans in Europe, most published surveys of EBLV1 infection in natural bat colonies have focused on other bat species (*5*,*12*). Data from serotine bats have been obtained by either serologic or direct detection techniques but not from both (*2*,*4*), as in this report.

Our recapture data show an absence of switching among colonies, even between colonies located only a few kilometers apart. This result indicates highly philopatric behavior among female bats, as has been confirmed in a recent mtDNA-based study of these colonies (J. Juste, unpub. data). This could be the cause of the different temporal distribution of the positive results observed in each colony, which suggests a pattern of independent endemic viral circulation different from the model, based on periodic epidemic waves of fast viral spreading proposed for the mouse-eared bat (*Myotis myotis*) (*12*).

The analysis of body condition index as a measure of physiologic condition gives additional evidence for mild or subclinical infection in the previously described long-term survival of EBLV1 RNA or antibody-positive bats (*2*,*4*,*5*,*12*). Similar body condition values between bats with oropharyngeal swabs that were positive for the virus and those that were negative could be interpreted as a recent virus infection for which no symptoms have developed. However, 1 lyssavirus-positive bat showed no viral RNA 3 years later. One bat even showed RNA in the oral cavity in consecutive samples separated by 1 week. Our findings suggest that asymptomatic EBLV1 RNA carriage may be common in serotine bats (*2*–*5*,*9*,*13*,*14*). In only a small subset of them does symptomatic neurologic infection progress to more severe body condition. However, most of these bats were captured while they were flying, and only 1 of those involved in an episode of human exposure had lost the ability to fly. This bat had the poorest body condition. The absence of Negri bodies and the moderate percentage of nerve cells affected found in the bat, captured while flying, suggest a subclinical process, as has been proposed for EBLV1 in *Rousettus aegyptiacus* (*6*,*14*). We cannot predict whether severe encephalitis was about to develop in these bats. The lack of pathogenicity could have arisen through long-standing coevolution between bats and viruses, as the phylogenetic data suggest (*15*). Nevertheless, additional work is necessary to establish whether the presence of EBLV1 RNA in the oral cavity is associated with the excretion of live virus.

In a similar study of *M. daubentonii* in the United Kingdom, only EBLV2 antibodies, but not viral RNA in the oropharyngeal cavity, were reported (*3*). Another study of the experimental transmission of Aravan, Khujand, and Irkut viruses to *E. fuscus* found oral excretion only in bats whose conditions were progressing to neurologic infection and death (*8*). These facts suggest differences between models of pathogenesis of lyssavirus infections in bats.

In summary, our findings indicate that natural maternity colonies of *E. isabellinus* behave as close communities in which EBLV1 independently circulates, which suggests an endemic pattern associated with asymptomatic or mild disease. Further studies are needed to 1) investigate the circumstances under which neurologic manifestations develop in bats (which lead to the abnormal behavior usually associated with human exposures) and 2) to establish the epidemiologic implications for public health of asymptomatic oral RNA carriage. The anthropophilic behavior of this bat species makes active surveillance highly recommended to predict risk factors that allow taking political decisions relating to public health.

## Supplementary Material

Appendix Table Number of European bat lyssavirus type 1-positive results for each colony of Eptesicus isabellinus bats (in gray fields), Spain.
