# Supplementary material for: Endemic Circulation of European Bat Lyssavirus Type 1 in Serotine Bats, Spain
Source: Emerg Infect Dis. 2008 Aug;14(8):1263–6. doi: 10.3201/1408.080068 (PMC2600403; doi:10.3201/1408.080068)
Supplement: Appendix Table — Number of European bat lyssavirus type 1-positive results for each colony of Eptesicus isabellinus bats (in gray fields), Spain. [file 08-0068_appT-s1.pdf]

Appendix Table. Number of European bat lyssavirus type 1–positive results for each colony of *Eptesicus isabellinus* bats (in gray fields), Spain

Colony	1998		1999		2000		2001		2002		2003		Total analyzed	
	RT-PCR	RFFIT	RT-PCR	RFFIT	RT-PCR	RFFIT	RT-PCR	RFFIT	RT-PCR	RFFIT	RT-PCR	RFFIT	RT-PCR	RFFIT
1	NT	NT	2	4	14	8	0	0	0	0	NT	NT	102	64
6	0	1	1	0	0	0	N.T	N.T	1	0	NT	NT	71	46
7	0	2	1	4	0	3	0	3	0	1	NT	NT	110	89
9	1	1	0	2	1	2	0	0	2	0	0	0	136	104
10	1	5	0	0	0	0	0	NT	0	0	0	0	118	68
11	0	0	1	1	0	0	0	NT	NT	NT	NT	NT	27	11
12	0	1	NT	NT	NT	NT	NT	NT	NT	NT	NT	NT	7	7
13	2	1	0	0	NT	NT	NT	NT	NT	NT	NT	NT	21	18
16	0	0	0	0	0	5	0	1	7	4	0	0	137	98
18	NT	NT	NT	NT	NT	NT	0	1	0	NT	0	NT	71	16

*RT-PCR, reverse transcription PCR; RFFIT, rapid fluorescent focus inhibition test; NT, not tested.
